# Chiropractic management of patients post-disc arthroplasty: eight case reports

**DOI:** 10.1186/1746-1340-18-7

**Published:** 2010-04-21

**Authors:** Julie O'Shaughnessy, Marc Drolet, Jean-François Roy, Martin Descarreaux

**Affiliations:** 1 Département de chiropratique, Université du Québec à Trois-Rivières, Trois-Rivières, Québec, Canada; 2 Private practice, Québec City, Canada; 3 Centre Hospitalier Universitaire de Québec, Hôpital St-François d'Assise, Québec, Québec, Canada

## Abstract

**Background:**

When conservative therapies for low back pain (LBP) are not effective, elective surgery may be proposed to these patients. Over the last 20 years, a new technology, disc replacement, has become increasingly popular because it is believed to maintain or restore the integrity of spinal movement and minimize the side-effects compared to fusion. Although disc replacement may relieve a patient from pain and related disability, soreness and stiffness of the lumbopelvic region seem to be common aftermaths of the surgery. This prospective case series was undertaken to identify and describe potential adverse events of lumbar spinal manipulation, a common therapy for low back pain, in a group of patients with symptoms after disc prostheses.

**Cases presentation:**

Eight patients who underwent lumbar spine total disc replacement were referred by an orthopaedic surgeon for chiropractic treatments. These patients had 1 or 2 total lumbar disc replacements and were considered stable according to the surgical protocol but presented persistent, post-surgical, non-specific LBP or pelvic pain. They were treated with lumbar spine side posture manipulations only and received 8 to 10 chiropractic treatments based on the clinical evolution and the chiropractor's judgment. Outcome measures included benign, self-limiting, and serious adverse events after low back spinal manipulative therapy. The Oswestry Disability Index, a pain scale and the fear avoidance belief questionnaire were administered to respectively assess disability, pain and fear avoidance belief about work and physical activity. This prospective case series comprised 8 patients who all had at least 1 total disc replacement at the L4/L5 or L5/S1 level and described persistent post-surgical LBP interfering with their daily activities. Commonly-reported side-effects of a benign nature included increased pain and/or stiffness of short duration in nearly half of the chiropractic treatment period. No major or irreversible complication was noted.

**Conclusions:**

During the short treatment period, no major complication was encountered by the patients. Moreover, the benign side-effects reported after lumbar spine manipulation were similar in nature and duration to those frequently experienced by the general population.

## Background

In industrialized countries, low back pain (LBP) is one of the most common heath problems, generating a great socioeconomic burden in the form of serious disability and work absenteeism [1-5]. Approximately three quarters of patients with chronic LBP consult health care professionals. They average 20 consultations with health care professionals per year [6]. It is well-known that non-specific LBP is the main reason for seeking chiropractic care, and spinal manipulation is one of the accepted and recommended therapies for LBP patients [7-9]. Sometimes, however, conservative therapies are not effective, and patients may be recommended for surgery.

In the United States, it has been estimated that between 6 and 7.5% of chronic LBP patients undergo spinal surgery [4,6]. The main reasons for surgical treatment of LBP are high levels of pain, disability and underlying pathology [10]. Patients with disc degeneration represent a large part of spinal surgery practice [11]. The incidence of disc degeneration increases with age, and roughly 100% of the population over 50 years of age will show some signs of lumbar spinal degeneration on radiographs, although the exact definition of "spinal degeneration" remains to be standardized [12]. In fact, in most cases, this degeneration may be a physiological part of spinal aging. Even though a precise diagnosis of chronic LBP is often impossible, spine degeneration is believed to be an important etiological factor in chronic LBP conditions [12-14]. Lumbar spinal degeneration includes central and/or lateral stenosis, facet joint osteoarthritis and disc deterioration [12]. Several theories have been put forward to explain the relationship between pain and disc degeneration: articular mechanoreceptor activation, functional segmental instability, inflammatory processes and chemical changes in discs have all been proposed as potential physiological causes of pain generation in degenerative disc disease [12,14,15].

For many years, vertebral fusion was considered to be the gold standard in surgery for disc degeneration [16]. The main goal of the procedure is to reduce spinal instability and limit abnormal segmental movement, consequently alleviating the pain. Despite the recognized utilization of this intervention, a reported long-term complication of fusion is the development of "adjacent segments degeneration", defined as the radiographic presence of disc deterioration adjacent to the surgically-treated disc [17]. Degeneration of adjacent segments is believed to be the consequence of increased motion of vertebrae above and below the fused segments. Such abnormal motion patterns are thought to heighten mechanical stresses at the discs and posterior elements, subsequently accelerating osteoarthritis and eliciting more pain in some cases [18-20]. Consequently, in patients with symptomatic adjacent segments, a second surgery may be necessary, often with limited success [18].

**Figure 1:**
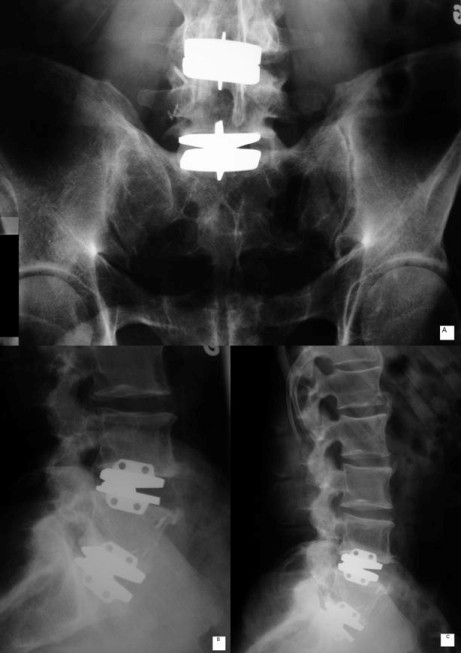
**Metal-on-metal disc prosthesis at L4-L5 and L5-S1**. a) antero-posterior radiograph, b) and c) lateral view.

In the last 20 years, a new technology, disc replacement, has become increasingly popular [21] but remains controversial [22]. This new procedure involves excision of the entire disc, which is replaced by a prosthesis (see Figure 1a, 1b and 1c). Surgery is performed by an anterior approach which leaves the posterior elements and muscles untouched [21,23,24]. Compared to fusion, disc replacement is believed to maintain or restore spinal movement integrity and reduce side-effects, such as blood loss, duration of surgery and hospital stay, the use of narcotic analgesics, and the development of adjacent segments degeneration [11,17,19,20,25-27]. Moreover, in comparison to fusion, disc replacement patients report a higher level of satisfaction and are more inclined to receive the same procedure if they again have the choice [27]. However, study of a comparative mathematical model representing a healthy spine showed in a fused model and a disc replacement higher biomechanical stresses at adjacent segments and at segments with disc prostheses [22]. In fact, research data on the functional outcomes of disc prostheses are limited, and those that have been published mainly involved cadaveric models.

Clinical outcome measures, such as the Oswestry Disability Index (ODI), the SF-36 health questionnaire, the visual analogue scale (VAS), medication prescription and patient satisfaction, have been investigated in several clinical trials to assess disc replacement efficacy and safety [19,27,28]. Although disc replacement may relieve a patient from symptoms (pain, disability and neurological deficits), in clinical practice, soreness and stiffness of the lumbopelvic region are frequently reported by patients after this surgery [29]. In a study assessing post-operative posterior joint pain (originating from the facets), it was found that 23.4% of disc prosthesis patients presented with post-operative posterior joint pain. The post-operative pain source was confirmed by a significant decrease in pain after fluoroscopic guided injection of combined local anaesthetic and corticosteroid [29]. These patients are often left with significant post-operative pain and may seek help from other health care professionals, such as manual therapists, for residual symptoms.

Spinal manipulation is one of the recommended conservative therapies for LBP [30-32]. The common side-effects of spinal manipulation in patients without surgery include local or radiating discomfort, tiredness, pain and soreness in approximately 50% of these patients. Usually, adverse events occur in the first 24 to 48 hours, are benign and resolve completely in a few days. Major complications, for which the overall prevalence is unknown, include increased pain from disc herniation (transient) or cauda equina syndrome (estimated to be less than 1/3,700,000 to 1/1,000,000 of lumbar manipulations) and are considered irreversible [8,33,34].

Since disc prostheses are believed to restore normal segmental range of motion and reduce potential residual instability, patients with total disc replacement could probably be manipulated similarly to patients with nonspecific LBP by standard side posture spinal manipulation to alleviate residual pain. This prospective case series was undertaken to identify and describe potential adverse events of lumbar spinal manipulation in a group of patients with disc prostheses. We hypothesized that, after disc replacement, patients with residual, chronic LBP would present spinal manipulation side-effects similar to those generally described in a chronic LBP population.

## Case presentation

### Patients

Eight patients who underwent lumbar total disc replacement were referred by an orthopaedic surgeon (JFR) for chiropractic treatments. These patients had 1 or 2 total lumbar disc replacements and were considered stable according to the surgical protocol. Prosthesis stability was analyzed on antero-posterior and lateral radiographs at 8 weeks and on lateral flexion and extension radiographs at 12 weeks. The prosthesis in all patients was a metal-on-metal *Maverick Lumbar Disc *Model (Medtronic Sofamor Danek, Inc., Memphis, TN, USA). However, despite this stability the patients involved in this study presented for chiropractic treatment with persistent, post-surgical, non-specific LBP or pelvic pain. Table 1 describes the baseline characteristics of these patients. The research protocol was approved by the research ethics review board of Université du Québec à Trois-Rivières (UQTR). All participants signed an informed consent form which included protocol information and the possible side-effects of spinal manipulation.

**Table 1:** Patient baseline information

Patients	Age (years)	Gender	Level(s) of prosthesis	Weeks post-surgery*
1	34	M	L5-S1	19
2	44	M	L4-L5 L5-S1	13
3	54	F	L4-L5	16
4	35	M	L5-S1	9
5	55	M	L4-L5 L5-S1	16
6	52	M	L5-S1	8
7	46	F	L5-S1	35
8	55	M	L4-L5 L5-S1	63

Mean (SD)	46.9 (8.7)	6M/2F	--	22.4 (18.4)

*Number of weeks between surgery and the first spinal manipulation.

### Clinical procedures

Patients were treated by 1 of the 2 practising chiropractors involved in the study, and only lumbar spine side posture manipulations were undertaken for standardization of the therapeutic procedure. After a brief neurological and orthopaedic examination was conducted to exclude the possibility of persisting neurological symptom, the segment to be manipulated by the chiropractor was identified using a combination of static and pain palpation (patients in prone position) regardless of disc prosthesis location. Both chiropractors performed the same sequence of examination, static and pain palpation prior to spinal manipulation. During the first chiropractic treatment, the patient was placed in a "preload" side posture position similar to the spinal manipulation procedure to evaluate if it was tolerated. Whenever the position did not yield any increase in lumbar pain level, a low-amplitude and high-velocity thrust was delivered at the painful segments, regardless of the disc prosthesis level. Patients were scheduled for 8 to 10 treatments at a frequency of twice a week [35]. The total number of treatments was based on their clinical status and the chiropractor's expertise.

### Outcome measures

Outcome measures included the assessment of benign, self-limiting, and serious adverse events following low back spinal manipulative therapy using a customized questionnaire completed at the beginning of each consultation. This qualitative questionnaire has not been validated and was developed for this study. The ODI and the Fear Avoidance Beliefs Questionnaire (FABQ) were administered to respectively assess disability and fear avoidance belief about work (FABQ I) and physical activity (FABQ II). The psychometric properties of the ODI and FABQ (French versions) have been assessed recently, and both questionnaires present moderate validity as well as good reproducibility [36,37]. Pain intensity was measured on a 1-5 intensity scale similar to the one proposed by Melzack [38]. These questionnaires were completed prior to the first chiropractic treatment, once a week and at the end of the treatment period. The frequencies of reported adverse events as well as mean values and standard deviations (SDs) for continuous variables were computed.

### Clinical results

Eight patients were included in this prospective case series. They all had at least 1 total disc replacement at the L4/L5 or L5/S1 level and described persistent post-surgical LBP interfering with daily activities. Mean baseline ODI score was 38.25 (2.0); mean pain score was 2.6 (1.1), whereas FABQ I (work) was 23.1 (16.4) and FABQ II (physical activity) was 13.3 (12.3). In all patients, pain was increased by manual, static or dynamic palpation of the lumbar segments.

The chiropractors were instructed to give between 1 to 10 lumbar spine manipulations. All of them were placed in a preload side posture position without any exacerbation of their pain. Spinal manipulation was considered successful when cavitation was audible or whenever the chiropractor was satisfied with the load applied and the resulting segmental motion (assessed by static and dynamic palpation).

The most common adverse event of a benign nature was a slight increase in short-duration pain (less than 12 hours) in almost half of treatments administered but two patients in the trial reported severe back and leg pains after the first treatment. (Table 2). The second most frequently-reported side-effect was light to moderate stiffness of short duration (12 to 24 hours). More serious but reversible side-effects included lower limb paresthesia in several patients (5 out of 8). However, these patients presented limb paresthesia before the intervention and exacerbation of their symptoms only lasted for a short time period (24 to 48 hours). They underwent periodic neurological examination, and no frank neurological deficit was found (diminished deep tendon reflexes, decreased motor strength). No major or irreversible complications were noted in these patients.

**Table 2:** Reported adverse events

Patients	Benign		Moderate		Severe		Severe/
	Back	Leg	Back	Leg	Back	Leg	irreversible
1					+	+	
2	+		+				
3			+	+			
4	+						
5			+				
6		+					
7					+	+	
8	+	+					

Frequency	3	2	3	1	2	2	0

As described in Table 3, symptoms improvement was characterized by a reduction of ODI scores in 6 patients, a decline of FABQ I scores in 4 patients and a decrease of FABQ II scores in 5 patients at the end of the treatment period.

**Table 3:** Clinical outcomes

Patients	Number of treatments	ODI (/100) Pre- Post-		Pain score (/5) Pre- Post-		FABQ I (/42) Pre- Post-		FABQ II (/24) Pre- Post-	
1	6	72	78	4	4	42	42	24	24
2	9	30	22	3	2	30	18	18	12
3	10	16	8	1	0	0	0	3	0
4	6	16	10	2	2	30	14	6	0
5	10	34	16	3	1	8	3	7	1
6	8	50	26	2	0	27	29	24	11
7	1	60	NA	4	4	42	NA	24	NA
8	9	28	8	2	1	6	0	0	0

Mean	7.4	38.3	24	2.6	1.8	23.1	15.1	13.3	6.9
SD	3.0	20.4	24.8	1.1	1.6	16.4	16.0	10.3	9.3

## Conclusions

The main objective of this study was to evaluate the potential adverse events of lumbar spine manipulations in disc replacement surgery patients. The most frequent side-effects reported were a slight increase in pain as well as minor to moderate lower back stiffness. Both side effects are frequently seen in non-surgical LBP patients after manipulation. In a recent study, Rubinstein [33] described post-spinal manipulation adverse events as being mild to moderate in intensity, with little or no influence on daily activities. He also showed that such events are brief, with spontaneous recovery and typically lasting no more than a few days. In the same article, it was mentioned that adverse events usually appear at the first treatment and are predictors of the worst prognosis. In a recent systematic review of safety of chiropractic intervention, the frequency of adverse events reported after a chiropractic intervention varied between 33 to 60.9%, regardless of treatment type and the patients' clinical presentation [39]. This analysis, however, did not investigate the methodological quality of the studies reviewed.

### Severe adverse reactions

In the present study, none of the patients had severe and irreversible reactions after spinal manipulation. Given the small sample size and, therefore, the possibility of severe adverse reactions to spinal manipulation in patients with disc prosthesis cannot be ruled out. Two patients in the trial reported severe back and leg pains after the first treatment. One of them was discharged, and the other received up to 6 treatments. For those two patients, additional evaluations were conducted and no frank neurological signs (deep tendon reflexes and motor evaluation), positive straight leg raised test (radiculopathy at >70°) or organic dysfunction were noted. One of these patients presented non-organic signs on physical examination, characterized by a "give away reaction" during motor function testing, as well as generalized overreaction to physical assessment. The other patient was referred back to the orthopaedic surgeon after a trial of lumbar spine manipulation. The orthopaedic surgeon administered a local lumbar facet joint injection of anaesthetic and anti-inflammatory agents that led to significant symptom reduction. It is noteworthy that these 2 patients had higher scores on the ODI questionnaire (72 and 60), on the pain scale (4) and on FABQ (I: 42 and II: 24) before the intervention. High levels of pain, disability and fear avoidance beliefs have all been identified as risk factors for the development of chronic LBP or associated with poor prognosis [3,40]. We could argue that similar risk factors are important determinants of post-surgery spinal manipulative treatments.

### Clinical considerations

Some patients will report residual, non-specific LBP after total disc replacement [29]. In cases where pain can be confirmed by semi-invasive procedures, various treatment options, such as local injection or manual therapy, can be considered. Although the purpose of this study was not to establish the clinical efficacy of spinal manipulation in patients with disc prosthesis, several clinical outcomes were assessed to better document the clinical presentation of each patient. In some cases, patients benefited from spinal manipulation and demonstrated clinically-significant (15-20%) improvement in pain and disability scores (see Table 3). In fact 5 patients presented a minimum clinically significant improvement in pain (9 mm) whereas 3 patients presented a minimum clinically significant improvement in disability (10 units) [41,42]. Obviously, because of its design, our study cannot answer the question as to whether these changes are specific effects of spinal manipulation, indeed they may have also occurred with a placebo or sham treatment. However, such clinical amelioration has frequently been reported in the literature, and lumbar spinal manipulation is usually deemed to be a valuable treatment option for short-term pain and disability improvement in chronic LBP populations [32,43]. In this study the limited improvements seen can best inform the development of a hypothesis to be tested.

### Limitations and recommendations

By nature, broad conclusions and generalization from case series studies are limited. Interpretation of the study is limited by its small sample size and the absence of long term follow-up. Other common and especially uncommon adverse reactions may not have been detected because of such limitations. However, standardization of outcome measures, evaluation and intervention during this trial may have restricted potential biases. Future investigations should address the standardization of adverse reaction outcomes across patients but most importantly across studies. Moreover, the current trial included patients who received a particular disc prosthesis, and not all prostheses may be suitable for spinal manipulation. In all cases, the origin of post surgical LBP must be thoroughly assessed and underlying pathologies (e.g., post-surgical hematoma) should be ruled out by a specialist before any manual therapy is initiated. Finally, collaboration with surgeons is essential to determine safety of spinal manipulation procedures in this group of patients.

### Future studies

Relationships between the best and worst outcomes of total disc replacement and several variables, such as gender, body mass index, occupation type, insurance type, surgery/prosthesis level and type, number of levels operated, smoker, back pain versus leg pain, previous surgery, radiographic placement, VAS scores, preoperative ODI scores and time off work before surgery, were studied recently [44,45]. Time off work before surgery was the only variable closely related to the prognosis of patients with total disc replacement and should therefore be assessed in long term follow-up studies.

In brief, although spinal manipulation only yielded modest pain and disability improvements during the short chiropractic treatment period, no major lasting complications were reported by the patients. Moreover, benign side-effects after lumbar spine manipulation were similar in nature and duration to those occurring in the general population. Clinical trials are needed to assess the safety and efficacy of spinal manipulation in patients with disc prostheses. Consultation with an orthopaedic surgeon should be essential prior to the initiation of spinal manipulative therapy in patient with disc prostheses until further research establishes effectiveness and safety.

### Consent

Written informed consent was obtained from the patients before the publication of this case series and any accompanying figure. A copy of the written consent forms was provided to the Editor-in-Chief of this journal.

## Competing interests

The authors declare that they have no competing interests.

## Authors' contributions

JOS and MDx were responsible for manuscript writing. JOS and MDt participated in the chiropractic treatments. JFR performed all clinical evaluations, surgery and postoperative follow-up. All authors have read and concur with the final manuscript. The article has not been submitted or published elsewhere.
